# The genetic determinants of oral diseases in Africa: The gaps should be filled

**DOI:** 10.3389/froh.2022.1017276

**Published:** 2022-10-11

**Authors:** Stephen M. Sudi, Salma Kabbashi, Imaan A. Roomaney, Marwa Aborass, Manogari Chetty

**Affiliations:** Craniofacial Biology, University of the Western Cape, Cape Town, South Africa

**Keywords:** genetics, africa, oral diseases, dental caries, periodontal diseasea, rare diseases

## Abstract

*Oral diseases* are a major health concern and are *among* the most prevalent *diseases globally*. This problem is becoming more prominent in the rapidly growing populations of Africa. It is well documented that Africa exhibits the most diverse genetic make-up in the world. However, little work has been conducted to understand the genetic basis of oral diseases in Africans. Oral health is often neglected and receives low prioritisation from funders and governments. The genetic determinants of highly prevalent oral diseases such as dental caries and periodontal disease, and regionally prevalent conditions such as oral cancer and NOMA, are largely under-researched areas despite numerous articles alluding to a high burden of these diseases in African populations. Therefore, this review aims to shed light on the significant gaps in research on the genetic and genomic aspects of oral diseases in African populations and highlights the urgent need for evidence-based dentistry, in tandem with the development of the dentist/scientist workforce.

## Introduction

The increase in the incidence of untreated caries of permanent teeth (1) in the 2019 Global Burden of Diseases, Injuries and Risk Factors Study (GBD) (2) is attributed to the population growth in sub-Saharan Africa. The same study reports the highest prevalence of severe periodontitis worldwide occurs in sub-Sahara Africa (3). Oral diseases and disorders (comprising dental caries, periodontal diseases, edentulism, and other oral disorders) are humankind's most prevalent chronic conditions. Oral diseases affect about 3.48 billion people and rank third globally in incidence (2). Africa, the second largest and second most populous continent, is home to a population of 1.4 billion (4), 3,000 different ethnic groups speaking more than 2,100 different languages (5). Most genetic diversity of human populations occurs in Africans (6). The sub-Saharan African region has the highest population growth rate of 2.8% globally. This population is expected to double between 2022 and 2050 and surpass 2 billion inhabitants by the late 2040s (4).

An estimated 480 million Africans suffer from oral disease. The World Health Organisation Africa Region has identified dental caries, periodontal diseases, oral cancers, Noma, oral manifestations of HIV and AIDS, oro-facial trauma and cleft lip and/or palate as priority oral diseases (7). Oral diseases share modifiable risk factors with cardiovascular disease (CVD), cancer, diabetes, and chronic respiratory diseases, the four most prevalent non-communicable diseases (NCDs) (8). These risk factors include an unhealthy diet high in sugar, tobacco use, and harmful use of alcohol (9). The burden of NCDs is increasing and will eclipse communicable, maternal, neonatal and nutritional (CMNN) diseases as the leading cause of mortality in sub-Saharan Africa by 2030 (10). Despite the interrelationship between oral diseases and other prevalent non-communicable diseases, a disjuncture in management approaches has been a consistent feature of oral and medical health services (11, 12). The oral health regional strategy for Africa aims to achieve better oral health as an integral part of NCDs (13). The recent global drive toward universal health coverage (UHC) by 2030, which addressed oral diseases (14) and the WHO Oral Health Strategy release (15), provide an opportune moment to spur global political commitments for oral health.

## Genetic studies and oral health

It is well documented that most genetic studies have been conducted on populations of European ancestry (16). The initiatives to characterize and understand genomic variations of human populations that followed the sequencing of human genome such as HapMap Project (17) and the 1,000 Genome Project (18), included representatives from African populations. These early initiatives further revealed the genomic complexity of African population and led to projects that focussed on sub-Saharan African such as the African Genome Variation Project (19) which revealed the influence of uncovered evidence of environment effects into the genetic susceptibility to conditions such as malaria, Lassa fever and trypanosomiasis (20). Several regional and country-based projects such as the Southern African Genome Project (21), the Ugandan Genome Resource (22) and the Nigeria 100 K Genome Project (23) also developed from these initiatives. An analysis of genome-wide association studies (GWAS) found that only 22% of the participants in these studies were of non-European origin with less than 4% of African and Latin American descent and from indigenous populations (24, 25). A study of only 16 South East Bantu speakers identified approximately 800,000 novel variants (26). Sherman et al., 2019, estimated that the African pan genome, has about 10% more abundance of DNA than the current human reference (27). In 2012, the Human Heredity and Health in Africa (H3Africa) program was launched with the aim to correct the dearth of genomics research in African continent (28). H3Africa initiative focused on capacity building through inter-continental collaborative projects, and the formation of African-based biorepositories and bioinformatics network, among other specific scientific goals (29). The Human Hereditary and Health Study in Africa (H3Africa) identified more than 3 million novel variants in a sample of 426 individuals (26, 30). In the H3Africa project, the genomics of craniofacial malformations in Africa were addressed through a multidisciplinary collaboration, the African Craniofacial Anomalies Network (AfriCRAN) (31).

Consequently, more than 100,000 participants were included in funded projects providing 50,000 genotyped samples and the identification of 26 core phenotypes. Furthermore, over 2000 workshops and meetings were held and around 700 papers were published (32).

Despite the notable achievements of H3Africa, common oral diseases are yet to be addressed in these initiatives; however, the complex genomic datasets established pave the way for projects that will ascertain the genetic determinants of prevalent oral diseases in Africa.

The paucity of studies on diverse genomes impedes our understanding of the human genome in health and disease (16), and results in the misclassification of variant pathogenicity in Africa. This situation limits the translation of genetic research into clinical practice, directly impacting patient management (18), public health policy, and ultimately exacerbates health inequalities (16). The 3 Million African Genome project (3MAG) was conceived in 2021 to sequence at least 3 million genomes carefully selected across Africa to cover ethnolinguistic and regional variation (33). The 3MAG project will improve the current situation and equip African scientists with knowledge that will enable informed approaches to a variety of public health challenges.

Explorative studies on genetic determinants of oral health and oral disease lag far behind that of other conditions. This problem is even more prominent in Africa, where only a few studies have focused on oral health and disease (34–39). The genetic determinants of highly prevalent oral diseases in African populations, such as dental caries and periodontal disease, and regionally prevailing conditions such as oral cancer and NOMA, are largely under-researched (40–42).

There are 54 countries in Africa with immense ethnic diversity. These populations differ in terms of wealth, educational accomplishment, living conditions, health systems and access to oral health services (42), hence, confirming the heterogeneity of the African population. These differences become critical as we move towards targeted healthcare (including oral health care).

This review aims at raising awareness of the genetic determinants of oral diseases and highlights research gaps in Africa. The review focuses on dental caries, periodontal disease, oral cancer, and rare diseases with craniofacial manifestations.

## Dental caries

Dental caries is a complex, chronic and multifactorial disease. It is mediated by the interplay between host factors, the microbial biofilm, a substrate that supports microbial cariogenicity and genetic influences (43, 44). The 2019 GBD report identified untreated caries of the permanent dentition, with an estimated 2 billion cases (95% uncertainty interval, 1.8 to 2.3 billion), as the most common health condition. Similarly, caries in deciduous teeth was reported as the most prevalent condition in children aged 0–14 years (1). A characterisation of the burden, trends and inequalities of untreated dental caries reported a lower prevalence of dental caries in the permanent dentition in developed countries (45). In this study, 64.6 million (95% CI, 64.4–64.9 million) and 62.9 million (62.8–63.1 million) cases of caries in the permanent and deciduous teeth, respectively, were attributed to sociodemographic inequality (44).

The incidence of untreated caries in the permanent dentition increased by 46.1% (95% uncertainty interval, 42%–50.3%) from 1990–2019 (1). It is important to note that the methodology used in the GBD studies employs spatiotemporal modelling analysis, leading to the underrepresentation of estimates from low-income settings (46).

The influence of genetic mechanisms on host factors, microbial biofilm and substrate has been suggested in different types of studies. In investigations involving twins (47), monozygotic but not dizygotic twins raised apart showed similarities in oral health status. The animal models studies involving rodents (48), suggestive quantitative locus traits (QLTs) were revealed on chromosomes 1, 2, 7 and 8. The variations in the amelogenin gene was described a factor in caries susceptibility study conducted in a Guatemalan- Mayan population (49). Recently, a genome-wide associations studies (GWAS) by Orlova et al.2019, suggested genetic differences in caries susceptibility and in potential genetic risk factors between African-Americans and Caucasians (50).

In a review of genetic and protein interactions in dental caries, Cavallari et al. (2019) provided an overview of 27 genes and genes—protein networks associated with protection or risk of dental caries. These were PRP1, PR, PA, MG1, MG2, AMELX, ENAM, TUFT1, KLK4, HLADR4, TAS1R3, TAS2R38, MBL2, MMP20, MMP2, MMP9, MMP13, GLUT2, TAS1R2, CA-VI, DEFB1, ALOX15, VDR-TAQI, MMP3, CA6, MUC5B, VDR-FOK and are related to enamel formation, development and mineralisation, host immune response and the composition of saliva (51).

Sub-Saharan Africa has subregions with high fluoride levels in groundwater sources, causing dental and skeletal fluorosis (52). Dental fluorosis is characterised by increased surface and subsurface enamel porosity (53). and is associated with increased dental caries (54, 55). Dental fluorosis presents with differing severity in individuals exposed to similar levels of fluoride intake, suggesting the influence of genetic factors and gene-environment interactions (56).

There is a scarcity of studies on the genetic and genetic-environment interaction in the dental caries disease process in Africans. Olatosi et al. described a replication of the signals for two single nucleotide polymorphisms previously reported for childhood caries in an investigation of the role of genetics in early childhood caries in Nigeria. The study reported different size effects for two loci in the Nigerian populations compared to a previous study on an American population (36). In a similar trend, a pilot GWAS assessing genes associated with dental caries in individuals of African descent described differences in the contributions of genetic variants to caries across racial groups (50). Investigating the genetic basis of dental caries and dental fluorosis among Africans is likely to improve the understanding of caries disease processes, identify risk groups, facilitate screening, guide priority setting in health care and strengthen disease prevention.

## Periodontal diseases

Periodontitis constitutes a major health concern due to its high prevalence and significant impact on general wellbeing (42). In fact, it has been estimated that 20%–50% of the global adult population has some degree of periodontitis (57). According to the latest GBD 2019 report, the highest prevalence of severe periodontitis was reported in sub-Saharan Africa (3).

In susceptible individuals, periodontitis develops from complex interactions between the dental biofilm microbiota, host immune-inflammatory response and environmental factors (58). An individual's susceptibility to periodontitis is dependent on their genetic background along with other risk factors such as poor oral hygiene and smoking (59). Cumulative evidence suggests that an association exists between periodontitis and different systemic diseases (60). For instance, pregnant women who are more susceptible to periodontal disease due to a hormonal surge, are more vulnerable to poor maternal and perinatal outcomes such as preeclampsia (61, 62). In addition, an association between periodontitis and chronic kidney disease has also been reported (63–65). Wahid et al. (2013) stated that published data support a bidirectional relationship between CKD and periodontal disease as patients with CKD also have a higher prevalence of periodontal disease (66).

Periodontitis, in essence, is a polygenic disease arising from variations in multiple gene loci in which each contributes to developing the clinical phenomena (67).

The genetic component of periodontitis appears to be more strongly associated with the aggressive phenotype (67). However, in the 2017 periodontal diseases classification scheme, the distinction between chronic and aggressive phenotypes is no longer justified (67). This is due to the current lack of evidence differentiating the pathophysiology between the two phenotypes (68). Therefore, periodontitis is currently classified as a single entity, “Periodontitis”, with the incorporation of staging and grading matrix for further diagnostic description (68, 69). In which, the staging vector demonstrates the disease severity and extent, and complexity of the management. While, the disease grade reflects the biological dimension of the infection and possible adverse effects on general health (70). The application of the new classification criteria led to the assignment of some previous phenotypes to a particular representative stage or grade. For instance, the previously classified aggressive periodontitis phenotype is currently under the grade C level (71).

A wealth of data suggests that genetic variations in host genes involved in modulation of the immune-inflammatory reaction to periodontitis, have a strong effect on disease susceptibility and development (72). Genetic variants may alter the modulatory proteins or their expression, which consequently results in innate and adaptive immunity alterations and may, therefore, govern disease outcome (72). Moreover, several studies highlight the variability in genetic susceptibility among different populations (54, 55).

Single nucleotide polymorphisms (SNPs) are common across the human population (>1%), however, their frequency varies significantly among various groups (67). Single nucleotide polymorphism (SNP) is the most common genetic variation investigated in the association with susceptibility to periodontitis (67). In particular, genes encoding for mediators involved in host immunity and metabolism such as cytokines and cell-surface receptors (72). Any dysregulation in genetic expression of these mediators might result in persistent destructive inflammation of the periodontium and the development of periodontitis (73). Accordingly, the association of particular SNPs with periodontitis differs among various populations and ethnic groups (67).

For instance, the gene which encodes for interlukin-1, one of the most important inflammatory cytokines involved in periodontitis pathogenesis, has been extensively investigated for its association with periodontitis susceptibility, however, findings have been contradictory (72, 74). A positive association between IL1A -889 C/T polymorphism and chronic periodontitis was reported in Caucasian, Asian, but not in the mixed Brazilian populations in a recently conducted large scale meta-analysis (75).

In Africa, there are more than two thousand distinct ethnic groups possessing the world's most diverse genetic makeup (76). It is likely that this genetic diversity will reflect a variation in susceptibility to periodontitis in Africa (76, 77). Nevertheless, little is known about periodontitis susceptibility among African populations (76).

## Oral cancer

The most pivotal malignancy of the oral cavity is squamous cell carcinoma (OSCC) arising from the mucosal epithelium. More than 90% of oral cancers are SCCs (78). Oral cancer ranks as the sixth most common cancer worldwide and as third in developing nations. However, there is evident under-reporting of cases in many countries in Africa due to a lack of cancer registries, cancer control programmes, modern health infrastructure, access to healthcare, finances, educational levels and existing religious and cultural beliefs (79–81).

In instances where African databases exist for the reporting of head and neck cancers, the incidence of cancers of the lip, tongue, oral cavity, and pharynx are often combined, and the oral cavity and oropharynx are most frequently reported among head and neck squamous cell carcinomas in Sub−Saharan Africa.

It is stated that the incidence of oral cancer is escalating in eastern and southern Africa. This is the result of increasing tobacco use, increased alcohol consumption, and traditional practices like chewing khat and tobacco, which are carcinogenic (82, 83). If detected early, oral cancers can be treated more easily.

The biologic behaviour of oral cancers, such as determining which would run an indolent or aggressive course, is an area requiring genetic and genomic investigations. This would contribute to their diagnosis and management. Diverse genetic, epigenetic, and environmental factors are involved in the pathogenesis oral SCC which has a poor prognosis (84). A systematic review assessing head and neck SCC in sub-Saharan Africa (45). found that none of the included studies reported on any genetic or genomic investigations.

The primary option for the management of oral SCC is determined by the stage of the disease and includes surgical resection, chemotherapy, radiotherapy, and immunotherapy (85). Despite advances in conventional therapy, several unfavourable consequences to therapy need to be further addressed. These include the fact that surgical resection may lead to long-term disfigurement and deformities that results in patients experiencing psychosocial stress and isolation. Radio- or chemo- therapies may cause significant toxicity or treatment resistance which ultimately compromise the quality of life of patients (85, 86).

Most OSCC are considered genetically unstable (87, 88). Frequently, chromosomal loss at 3p, 8p, 9p, 17p and gains at 3q and 11q have been reported (89). When these changes extend for some distance from the clinical lesion, the clinical phenomenon of field cancerisation is described (90). Genes that have often been reported to have a role in the development of OSCC are TP53, CDKN2A, PTEN, HRAS and PIK3CA (89, 91, 92). The evidence for inherited genetic susceptibility for the development of OSCC has been difficult to source.

The effectiveness of the various current therapeutic modalities is reliant on the genetic and mutational profile of the tumour as this contributes to the oncogenic potential of the lesion. This type of targeted therapy is directed at these genetic modifications and forms the basis of precision medicine (84, 93).

Despite considerable advancement which has been made in the management of oral cancer, its molecular heterogeneity still needs to be investigated in Africa. The molecular basis of the tumour also requires scrutiny in order to elucidate instances of resistance to therapeutic measures (84).

## Rare diseases

Congenital defects and hereditary syndromes affecting the craniofacial complex are amongst the most common health problems globally (31, 94). These conditions significantly impact the quality of life of those affected and exacerbate health inequalities, particularly in low-resourced LMICs (31). Due to a lack of available data, rare diseases in Africa remain unquantified (95). Current estimates place the number of African people directly affected by rare diseases (RD) at 50 million (96, 97). The number of people indirectly affected is much higher due to the high economic and societal burden of RD (98, 99). At least 72% of RDs are believed to be of genetic origin (97).

Oral and facial clefts (OFCs) serve as a good model for studying the etiology, treatment and prevention of congenital disabilities (100). The 2022 global incidence report of cleft lip/ palate (CL/P) found that CL/P prevalence is highest in Asian (∼1/500) and American populations and significantly lower in African populations (∼1/2,500) (101). There has been increasing interest in OFC studies on the continent (34, 35, 37, 38, 100). Butali et al. (2019) conducted a study on OFCs with 3,178 participants from Ghana, Nigeria and Ethiopia (37). They were able to identify two novel loci with genome-wide significance and were able to confirm previously reported loci from GWAS studies from other populations. However, the authors identified the need to perform whole genome studies to provide a more comprehensive analysis of all classes of variants, including rare variants. Identifying variants protective of OFCs may open the possibility of therapies in the future (38).

There have been few genotype-phenotype correlation studies in large cohorts of patients with RDs in Africa. The variability in phenotypic expression, high genetic heterogeneity and low mutation frequency were noted as challenges to establishing consistent genotype-phenotype correlations (96). A study in Sub-Saharan Africa found that geneticists had difficulty diagnosing Williams-Beuren Syndrome based on facial dysmorphic features (102, 103). Another study found that an artificial intelligence (AI) based tool was only able to classify 35% of Congolese participants with Down's Syndrome compared to 80% of Belgian participants, prior to specific training (104).

Recent studies in South Africa assessed osteogenesis imperfecta (OI) type 3, which has an unusually high frequency in the Black Southern African populations (105) Out of 91 patients with a confirmed phenotypic diagnosis of OI type 3, 45% of affected individuals had a pathogenic variant of the *FKBP10* gene (105). Interestingly, the individuals with a homozygous mutation in the *FKBP10* gene have clinically unaffected teeth yet exhibited radiographic features of dentinogenesis imperfecta to varying degrees (106). Another study evaluating the physical features of 125 individuals with Noonan syndrome found that Black Africans had the most distinctive features (107) however, the molecular detection rate in this study was only 31.2% compared to the expected >70% using whole exome sequencing (WES). This suggests that the pathogenic variants are likely in genes that were not investigated. These studies are important as they enable a more accurate clinical diagnosis of patients, especially where molecular studies are unavailable.

## Epilogue

The diverse genetic and genomic heterogeneity of Africans has been the result of ancient migration patterns and adaptive pressures on the human genome. This resulted in evolutionary events, which spilt the human population into five distinct groups: southern Khoi-San, northern Khoi-San, central African hunter-gatherers, West Africans, and East Africans. A subset migrated out of Africa and is now identified as the out-of-Africa population (108, 109). Hence, the continent of Africa can be said to be the repository of human genomic diversity and can consequently serve as the reference resource for understanding the role of genomics in human health and disease.

It is apparent that Africa is home to a huge burden of oral diseases, with limited genetic and genomic research. In the era of targeted approaches to the management of human diseases, novel discoveries in the biomedical sciences are redefining the conduct of research and improving oral health by translating these findings into clinical application (93).

It has become imperative for the African oral health fraternity to prepare and arm itself with personnel who are equipped to investigate and interpret knowledge obtained from genomics, genetic sequencing, transcriptomics and proteomics, molecular profiling and bioinformatic data. This is essential in order to improve the management of oral health diseases and disorders in the era of targeted therapy, and will facilitate the development of disease management protocols aimed at African patients and conditions (110).

Roberts et al. (2020), proposed the inclusion of adequate training in the genetics of oral diseases in undergraduate and postgraduate dental programmes for African universities. With adequate skillsets the trained dentists will have an interest in following a career in genetic and genomic oral health research. This would enhance networking among African dental researchers and lead to improved dental research output and evidence based dentistry across the continent (111).

However, the high cost of biomedical infrastructure, laboratory set-up, project establishment and remuneration, has prevented many with an interest in biomedical research from engaging in genetic and genomic research. The genome sequencing technology is undergoing an evolutionary growth with the entry of new companies and the introduction of new techniques, which promise a significant reduction in costs (112). Joint efforts among African countries to increase funding and remuneration, as well as collaboration among institutions, will facilitate growth and prevent wasteful expenditure that may occur through duplication of these efforts.

In the current scenario where low- and middle-income countries (LMICs), most of which are in Africa, are left out of the advances in genomic technologies, The WHO Science Council of Experts released a report on accelerating access to genomics for global health. This report recommends approaches based on four themes, implementation, advocacy collaboration and associated ethical legal and social issues. The WHO Science Council proposed the establishment of a genomics committee to assist in rendering genomic technology affordable in LMICs by engaging various stakeholder and commercial entities (113).

Thus, there is an urgent and pressing need for oral health researchers and policy makers to develop and participate in translational clinical research that will accelerate targeted scientific breakthroughs in the management of oral diseases in Africa.

## References

[1] Vos T Lim SS Abbafati C Abbas KM Abbasi M Abbasifard M Global burden of 369 diseases and injuries in 204 countries and territories, 1990–2019: a systematic analysis for the Global Burden of Disease Study 2019. Lancet. (2020) [cited 2022 Jul 25] 396(10258):1204–22. 10.1016/S0140-6736(20)30925-9 33069326 PMC7567026

[2] Oral disorders — Level 3 cause. Institute for Health Metrics and Evaluation. (2020) [cited 2022 Jul 24]. Available from: https://www.healthdata.org/results/gbd_summaries/2019/oral-disorders-level-3-cause

[3] Wu L Zhang S Zhao L Ren Z Hu C. Global, regional, and national burden of periodontitis from 1990 to 2019: results from the Global Burden of Disease study 2019. J Periodontol. (2022).Available from: https://onlinelibrary.wiley.com/doi/abs/10.1002/JPER.21-0469 10.1002/JPER.21-0469 35305266

[4] United Nations Department of Economic and Social Affairs, Population Division. World Population Prospects 2022, Summary of Results.pdf. United Nations Publication. (2022). Report No.: UN DESA/POP/2022/TR/NO. 3

[5] Obadina E. Ethnic groups in Africa. Broomall, PA: Mason Crest (2014). 173 p

[6] Ramsay M Tiemessen CT Choudhury A Soodyall H. Africa: the next frontier for human disease gene discovery? Hum Mol Genet. (2011) 20(R2):R214–20. 10.1093/hmg/ddr401 21908518

[7] World Health Organization. Regional office for Africa. Promoting Oral Health in Africa: Prevention and control of oral diseases and noma as part of essential noncommunicable disease interventions. Brazzaville: World Health Organization. Regional Office for Africa (2016) [cited 2022 Aug 4]. Available from: https://apps.who.int/iris/handle/10665/205886

[8] Peres MA Macpherson LMD Weyant RJ Daly B Venturelli R Mathur MR Oral diseases: a global public health challenge. Lancet. (2019) [cited 2022 Jul 24] 394(10194):249–60. 10.1016/S0140-6736(19)31146-8 31327369

[9] Petersen PE Bourgeois D Ogawa H Estupinan-Day S Ndiaye C. The global burden of oral diseases and risks to oral health. Bull World Health Organ. (2005) 83:661–9. Available from: https://www.researchgate.net/publication/7554914_The_Global_Burden_of_Oral_Diseases_and_Risks_to_Oral_Health 16211157 PMC2626328

[10] Gouda HN Charlson F Sorsdahl K Ahmadzada S Ferrari AJ Erskine H Burden of non-communicable diseases in sub-Saharan Africa, 1990–2017: results from the Global Burden of Disease Study 2017. Lancet Glob Health. (2019) [cited 2022 Jul 24] 7(10):e1375–87. 10.1016/S2214-109X(19)30374-2 31537368

[11] Crosser D. Oral health: oft overlooked. Lancet Child Adolesc Health. (2019) [cited 2022 Jul 24] 3(10):663. 10.1016/S2352-4642(19)30275-5 31526741

[12] Huang YK Chang YC. Oral health: the first step to sustainable development goal 3. J Formos Med Assoc. (2022) [cited 2022 Jul 24] 121(7):1348–50. 10.1016/j.jfma.2021.10.018 34732302

[13] Regional Committee for Africa 66. Regional oral health strategy 2016–2025: addressing oral diseases as part of noncommunicable diseases: report of the Secretariat. World Health Organization. Regional Office for Africa (2016) [cited 2022 Jul 24]. Report No.: AFR/RC66/5. Available from: https://apps.who.int/iris/handle/10665/250994

[14] Wang TT Mathur MR Schmidt H. Universal health coverage, oral health, equity and personal responsibility. Bull World Health Organ. (2020) [cited 2022 Jul 24] 98(10):719–21. 10.2471/BLT.19.247288 33177761 PMC7652557

[15] Benzian H Guarnizo-Herreño CC Kearns C Muriithi MW Watt RG. The WHO global strategy for oral health: an opportunity for bold action. The Lancet. (2021) [cited 2022 Jul 24] 398(10296):192–4. 10.1016/S0140-6736(21)01404-5 34174182

[16] Sirugo G Williams SM Tishkoff SA. The missing diversity in human genetic studies. Cell. (2019) 177(1):26–31. 10.1016/j.cell.2019.02.048 30901543 PMC7380073

[17] Gibbs RA Belmont JW Hardenbol P Willis TD Yu F Yang H The international HapMap project. Nature. (2003) 426(6968):789–96. 10.1038/nature02168 14685227

[18] Via M Gignoux C Burchard EG. The 1000 genomes project: new opportunities for research and social challenges. Genome Med. (2010) 2(1):3. 10.1186/gm124 20193048 PMC2829928

[19] Gurdasani D Carstensen T Tekola-Ayele F Pagani L Tachmazidou I Hatzikotoulas K The african genome variation project shapes medical genetics in Africa. Nature. (2014) 517(7534):327–32. Available from: https://www.nature.com/articles/nature13997 25470054 10.1038/nature13997 PMC4297536

[20] Adama. The African Genome Project—Why is it important ?. website. (2021) [cited 2022 Sep 12]. Available from: https://www.thecatalystinme.com/single-post/the-african-genome-project-why-is-it-important

[21] Pepper MS. Launch of the Southern African human genome programme. SAMJ South Afr Med J. (2011) 101(5):287–8. 10.7196/SAMJ.4860 21837864

[22] Gurdasani D Carstensen T Fatumo S Chen G Franklin CS Prado-Martinez J Uganda genome resource enables insights into population history and genomic discovery in Africa. Cell. (2019) 179(4):984–1002.e36. 10.1016/j.cell.2019.10.004 31675503 PMC7202134

[23] https://www.nature.com/articles/s41588-022-01071-6.

[24] Mills MC Rahal C. A scientometric review of genome-wide association studies. Commun Biol. (2019) 2:9. 10.1038/s42003-018-0261-x 30623105 PMC6323052

[25] D’Angelo CS Hermes A McMaster CR Prichep E Richer É van der Westhuizen FH Barriers and considerations for diagnosing rare diseases in indigenous populations. Front Pediatr. (2020) [cited 2022 Jul 23] 8. Available from: https://www.frontiersin.org/articles/10.3389/fped.2020.579924 10.3389/fped.2020.579924 PMC7767925 33381478

[26] Choudhury A Ramsay M Hazelhurst S Aron S Bardien S Botha G Whole-genome sequencing for an enhanced understanding of genetic variation among South Africans. Nat Commun. (2017) 8(1):1–12. 10.1038/s41467-016-0009-6 29233967 PMC5727231

[27] Sherman RM Forman J Antonescu V Puiu D Daya M Rafaels N Assembly of a pan-genome from deep sequencing of 910 humans of African descent. Nat Genet. (2019) 51(1):30–5. 10.1038/s41588-018-0273-y 30455414 PMC6309586

[28] H3Africa consortium. Enabling the genomic revolution in Africa. Science. (2014) 344(6190):1346–8. 10.1126/science.1251546 24948725 PMC4138491

[29] Ramsay M. Growing genomic research on the African continent: the H3Africa Consortium. S Afr Med J. (2015) 105:1016. 10.7196/SAMJ.2015.v105i12.10281 26792157

[30] Pereira L Mutesa L Tindana P Ramsay M. African genetic diversity and adaptation inform a precision medicine agenda. Nat Rev Genet. (2021) 22(5):284–306. 10.1038/s41576-020-00306-8 33432191

[31] Butali A Mossey P Tiffin N Adeyemo W Eshete M Mumena C Multidisciplinary approach to genomics research in Africa: the AfriCRAN model. Pan Afr Med J. (2015) 21:229. Available from: https://www.ncbi.nlm.nih.gov/pmc/articles/PMC4607986/26523171 10.11604/pamj.2015.21.229.7380 PMC4607986

[32] Human Heredity and Health in Africa (H3Africa) resources and achievements endure beyond its end—Fogarty International Center @ NIH. Fogarty International Center. [cited 2022 Sep 11]. Available from: https://www.fic.nih.gov:443/News/GlobalHealthMatters/march-april-2022/Pages/human-heredity-health-h3-africa-achievements-endure.aspx

[33] Wonkam A. Sequence three million genomes across Africa. Nature. (2021) 590:209–11. 10.1038/d41586-021-00313-7 33568829 PMC9979155

[34] https://www.iadr.org/about/news-reports/press-releases/african-cleft-gwas-signal-replication-independent-african-cohort.

[35] Mukhopadhyay N Feingold E Moreno-Uribe L Wehby G Valencia-Ramirez LC Muñeton CPR Genome-wide association study of non-syndromic orofacial clefts in a multiethnic sample of families and controls identifies novel regions. Front Cell Dev Biol. (2021) [cited 2022 Jul 21] 9:621482. 10.3389/fcell.2021.621482 33898419 PMC8062975

[36] Olatosi OO Li M Alade AA Oyapero A Busch T Pape J Replication of GWAS significant loci in a sub-Saharan African Cohort with early childhood caries: a pilot study. BMC Oral Health. (2021) 21(1):274. 10.1186/s12903-021-01623-y 34016088 PMC8139096

[37] Butali A Mossey PA Adeyemo WL Eshete MA Gowans LJJ Busch TD Genomic analyses in African populations identify novel risk loci for cleft palate. Hum Mol Genet. (2019) 28(6):1038–51. 10.1093/hmg/ddy402 30452639 PMC6400042

[38] Gowans LJJ Adeyemo WL Eshete M Mossey PA Busch T Aregbesola B Association studies and direct DNA sequencing implicate genetic susceptibility loci in the etiology of nonsyndromic orofacial clefts in sub-saharan african populations. J Dent Res. (2016) 95(11):1245–56. 10.1177/0022034516657003 27369588 PMC5076758

[39] Eshete MA Liu H Li M Adeyemo WL Gowans LJJ Mossey PA Loss-of-function GRHL3 variants detected in african patients with isolated cleft palate. J Dent Res. (2018) 97(1):41–8. 10.1177/0022034517729819 28886269 PMC5755809

[40] Chikte U Pontes CC Karangwa I Kimmie-Dhansay F Erasmus R Kengne AP Dental caries in a South African adult population: findings from the Cape Town Vascular and Metabolic Health Study. Int Dent J. (2020) 70(3):176–82. 10.1111/idj.12538 31808148 PMC9379168

[41] Chidzonga MM Carneiro LC Kalyanyama BM Kwamin F Oginni FO. Determinants of oral diseases in the African and Middle East region. Adv Dent Res. (2015) 27(1):26–31. 10.1177/0022034515581645 26101337

[42] Abid A Maatouk F Berrezouga L Azodo C Uti O El-Shamy H Prevalence and severity of oral diseases in the Africa and Middle East Region. Adv Dent Res. (2015) 27(1):10–7. 10.1177/0022034515582062 26101335

[43] Vieira AR. Genetics and caries—perspectives. Braz Oral Res. (2012) [cited 2022 Jul 25] 26(Suppl. 1):7–9. 10.1590/S1806-83242012000700002 23318738 PMC3558839

[44] Pitts NB Twetman S Fisher J Marsh PD. Understanding dental caries as a non-communicable disease. Br Dent J. (2021) [cited 2022 Jul 25] 231(12):749–53. 10.1038/s41415-021-3775-4 34921271 PMC8683371

[45] Wen PYF Chen MX Zhong YJ Dong QQ Wong HM. Global burden and inequality of dental caries, 1990 to 2019. J Dent Res. (2022) [cited 2022 Jul 25] 101(4):392–9. 10.1177/00220345211056247 34852668

[46] Murray CJL Aravkin AY Zheng P Abbafati C Abbas KM Abbasi-Kangevari M Global burden of 87 risk factors in 204 countries and territories, 1990–2019: a systematic analysis for the Global Burden of Disease Study 2019. Lancet. (2020) [cited 2022 Jul 25] 396(10258):1223–49. 10.1016/S0140-6736(20)30752-2 33069327 PMC7566194

[47] Boraas JC Messer LB Till MJ. A genetic contribution to dental caries, occlusion, and morphology as demonstrated by twins reared apart. J Dent Res. (1988) [cited 2022 Jul 26] 67(9):1150–5. 10.1177/00220345880670090201 3165997

[48] Nariyama M Shimizu K Uematsu T Maeda T. Identification of chromosomes associated with dental caries susceptibility using quantitative trait locus analysis in mice. Caries Res. (2004) 38(2):79–84. 10.1159/000075929 14767162

[49] Deeley K Letra A Rose EK Brandon CA Resick JM Marazita ML Possible association of amelogenin to high caries experience in a guatemalan-mayan population. Caries Res. (2008) [cited 2022 Jul 26] 42(1):8–13. 10.1159/000111744 18042988 PMC2814012

[50] Orlova E Carlson JC Lee MK Feingold E McNeil DW Crout RJ Pilot GWAS of caries in African-Americans shows genetic heterogeneity. BMC Oral Health. (2019) [cited 2022 Jul 25] 19(1):215. 10.1186/s12903-019-0904-4 31533690 PMC6751797

[51] Cavallari T Arima LY Ferrasa A Moysés SJ Tetu Moysés S Hirochi Herai R Dental caries: genetic and protein interactions. Arch Oral Biol. (2019) [cited 2022 Jul 25] 108:104522. 10.1016/j.archoralbio.2019.104522 31476523

[52] Chowdhury A Adak MK Mukherjee A Dhak P Khatun J Dhak D. A critical review on geochemical and geological aspects of fluoride belts, fluorosis and natural materials and other sources for alternatives to fluoride exposure. J Hydrol. (2019) [cited 2022 Aug 14] 574:333–59. 10.1016/j.jhydrol.2019.04.033

[53] Aoba T Fejerskov O. Dental fluorosis: chemistry and biology. Crit Rev Oral Biol Med Off Publ Am Assoc Oral Biol. (2002) 13(2):155–70. 10.1177/154411130201300206 12097358

[54] Grobler SR Louw AJ Van Kotze TJ. Dental fluorosis and caries experience in relation to three different drinking water fluoride levels in South Africa. Int J Paediatr Dent. (2001) [cited 2022 Jul 26] 11(5):372–9. 10.1046/j.0960-7439.2001.00293.x 11572269

[55] Wondwossen F Astrom AN Bjorvatn K Bardsen A. The relationship between dental caries and dental fluorosis in areas with moderate- and high-fluoride drinking water in Ethiopia. Community Dent Oral Epidemiol. (2004) [cited 2022 Jul 26] 32(5):337–44. 10.1111/j.1600-0528.2004.00172.x 15341618

[56] Abbasoglu Z Dalledone M Wambier LM Pecharki G Baratto-Filho F Andrades KMR Single nucleotide polymorphism rs4284505 in microRNA17 and risk of dental fluorosis. Acta Odontol Scand. (2020) [cited 2022 Jul 26] 78(6):463–6. 10.1080/00016357.2020.1786600 32619376

[57] Nazir MA. Prevalence of Periodontal disease, Its Association with Systemic Diseases and Prevention. (2017). Available from: https://www.ncbi.nlm.nih.gov/pmc/articles/PMC5426403/pdf/IJHS-11-72.pdf PMC5426403 28539867

[58] Zhang S Yu N Arce RM. Periodontal inflammation: integrating genes and dysbiosis. Periodontol 2000. (2019) 82(1):129–42. 10.1111/prd.12267 PMC6924568 31850627

[59] Tettamanti L. Genetic susceptibility and periodontal disease: a retrospective study on a large Italian sample. Oral Implantol. (2017) 10(1):20. 10.11138/orl/2017.10.1.020 PMC5516423 28757932

[60] Albandar JM Susin C Hughes FJ. Manifestations of systemic diseases and conditions that affect the periodontal attachment apparatus: case definitions and diagnostic considerations. J Periodontol. (2018) 89(Suppl. 1):S183–S203. 10.1002/JPER.16-0480 29926941

[61] Bi WG Emami E Luo ZC Santamaria C Wei SQ. Effect of periodontal treatment in pregnancy on perinatal outcomes: a systematic review and meta-analysis. J Matern Fetal Neonatal Med. (2019):1–10. 10.1080/14767058.2019.1678142 31630597

[62] Tettamanti L. Pregnancy and periodontal disease: does exist a two-way relationship? Oral Implantol. (2017) 10(2):112. 10.11138/orl/2017.10.2.112 PMC5965070 29876036

[63] Dye BA Choudhary K Shea S Papapanou PN. Serum antibodies to periodontal pathogens and markers of systemic inflammation. J Clin Periodontol. (2005) 32(12):1189–99. 10.1111/j.1600-051X.2005.00856.x 16268994

[64] Cazzavillan S Ratanarat R Segala C Corradi V de Cal M Cruz D Inflammation and subclinical infection in chronic kidney disease: a molecular approach. Blood Purif. (2007) 25(1):69–76. 10.1159/000096401 17170541

[65] Stenvinkel P. New insights on inflammation in chronic kidney disease-genetic and non-genetic factors. Nephrol Ther. (2006) 2(3):111–9. 10.1016/j.nephro.2006.04.004 16890135

[66] Wahid A Chaudhry S Ehsan A Butt S Ali Khan A. Bidirectional relationship between chronic kidney disease / periodontal disease. Pak J Med Sci. (2013) 29(1):211–5. Available from: https://www.ncbi.nlm.nih.gov/pmc/articles/PMC3809193/24353542 10.12669/pjms.291.2926 PMC3809193

[67] Gonçalves PF Harris TH Elmariah T Aukhil I Wallace MR Shaddox LM. Genetic polymorphisms and periodontal disease in populations of African descent: a review. J Periodontal Res. (2017) 53(2):164–73. 10.1111/jre.12505 29105764 PMC5912940

[68] Tonetti MS Greenwell H Kornman KS. Staging and grading of periodontitis: framework and proposal of a new classification and case definition. J Periodontol. (2018) 89(Suppl. 20):S159–72. 10.1002/JPER.18-0006 29926952

[69] Caton JG Armitage G Berglundh T Chapple ILC Jepsen S Kornman KS A new classification scheme for periodontal and peri-implant diseases and conditions—introduction and key changes from the 1999 classification. J Periodontol. (2018) 89(Suppl. 20):S1–8. Available from: https://onlinelibrary.wiley.com/doi/abs/10.1111/jcpe.12935 29926946 10.1002/JPER.18-0157

[70] Kornman KS Papapanou PN. Clinical application of the new classification of periodontal diseases: ground rules, clarifications and “gray zones.”. J Periodontol. (2020) 91(3):352–60. 10.1002/JPER.19-0557 31891194

[71] Shaddox LM Morford LA Nibali L. Periodontal health and disease: the contribution of genetics. Periodontol 2000. (2020) 85(1):161–81. 10.1111/prd.12357 33226705 PMC13404626

[72] Uslu M Toy V. Do genetic polymorphisms affect susceptibility to periodontal disease? A literature review. Niger J Clin Pract. (2019) 22(4):445. 10.4103/njcp.njcp_462_18 30975946

[73] Dosseva-Panova V Mlachkova A Popova C. Gene polymorphisms in periodontitis. Overview. Biotechnol Biotechnol Equip. (2015) 29(5):834–9. 10.1080/13102818.2015.1056230

[74] Ramadan DE Hariyani N Indrawati R Ridwan RD Diyatri I. Cytokines and chemokines in periodontitis. Eur J Dent. (2020) 14(03):483–95. 10.1055/s-0040-1712718 32575137 PMC7440949

[75] da Silva MK de Carvalho ACG Alves EHP da Silva FRP dos Pessoa LS Vasconcelos DFP. Genetic factors and the risk of periodontitis development: findings from a systematic review composed of 13 studies of meta-analysis with 71,531 participants. Int J Dent. (2017) 2017:1–9. 10.1155/2017/1914073 PMC5424192 28529526

[76] Baelum V Scheutz F. Periodontal diseases in Africa. Periodontol 2000. (2002) 29(1):79–103. 10.1034/j.1600-0757.2002.290105.x 12102704

[77] Tishkoff SA Williams SM. Genetic analysis of African populations: human evolution and complex disease. Nat Rev Genet. (2002) 3(8):611–21. 10.1038/nrg865 12154384

[78] Gupta N Gupta R Acharya AK Patthi B Goud V Reddy S Changing trends in oral cancer—a global scenario. Nepal J Epidemiol. (2016) 6(4):613–9. 10.3126/nje.v6i4.17255 28804673 PMC5506386

[79] Korir A Okerosi N Ronoh V Mutuma G Parkin M. Incidence of cancer in Nairobi, Kenya (2004–2008). Int J Cancer. (2015) 137(9):2053–9. 10.1002/ijc.29674 26139540

[80] Faggons C Mabedi C Shores C Gopal S. Review: head and neck squamous cell carcinoma in sub-Saharan Africa. Malawi Med J. (2015) 27(3):79–87. 10.4314/mmj.v27i3.2 26715951 PMC4688867

[81] Gakunga R Parkin DM, African Cancer Registry Network. Cancer registries in Africa 2014: a survey of operational features and uses in cancer control planning. Int J Cancer. (2015) 137(9):2045–52. 10.1002/ijc.29668 26135162

[82] Pacella R Urban M Sitas F Carrara H Sur R Hale M Risk factors for oesophageal, lung, oral and laryngeal cancers in Black South Africans. Br J Cancer. (2002) 86:1751–6. 10.1038/sj.bjc.6600338 12087462 PMC2375408

[83] Al-Hebshi NN Alharbi FA Mahri M Chen T. Differences in the bacteriome of smokeless tobacco products with different oral carcinogenicity: compositional and predicted functional analysis. Genes (Basel). (2017) 8(4):E106. 10.3390/genes8040106 28333122 PMC5406853

[84] Usman S Jamal A Teh MT Waseem A. Major molecular signaling pathways in oral cancer associated with therapeutic resistance. Front Oral Health. (2021) [cited 2022 Aug 4] 1. 10.3389/froh.2020.603160 35047986 PMC8757854

[85] Beaudoin PL Anchouche S Gaffar R Guadagno E Ayad T Poenaru D. Barriers in access to care for patients with head and neck cancer in resource-limited settings: a systematic review. JAMA Otolaryngol– Head Neck Surg. (2020) 146(3):291–7. 10.1001/jamaoto.2019.4311 31944228

[86] Coutts KA Seedat J Vlok E. The management of head and neck cancer in Africa. What lessons can be learned from African literature? South Afr J Oncol. (2022) 6:3. Available from: https://sajo.org.za/index.php/sajo/article/view/204

[87] Bhattacharya A Roy R Snijders AM Hamilton G Paquette J Tokuyasu T Two distinct routes to oral cancer differing in genome instability and risk for cervical node metastasis. Clin Cancer Res Off J Am Assoc Cancer Res. (2011) 17(22):7024–34. 10.1158/1078-0432.CCR-11-1944 PMC3226754 22068658

[88] Pickering CR Zhang J Yoo SY Bengtsson L Moorthy S Neskey DM Integrative genomic characterization of oral squamous cell carcinoma identifies frequent somatic drivers. Cancer Discov. (2013) 3(7):770–81. 10.1158/2159-8290.CD-12-0537 23619168 PMC3858325

[89] Lawrence MS Sougnez C Lichtenstein L Cibulskis K Lander E Gabriel SB Comprehensive genomic characterization of head and neck squamous cell carcinomas. Nature. (2015) 517(7536):576–82. 10.1038/nature14129 25631445 PMC4311405

[90] Braakhuis BJM Tabor MP Kummer JA Leemans CR Brakenhoff RH. A genetic explanation of Slaughter’s concept of field cancerization: evidence and clinical implications. Cancer Res. (2003) 63(8):1727–30. Available from: https://aacrjournals.org/cancerres/article/63/8/1727/511142/A-Genetic-Explanation-of-Slaughter-s-Concept-of 12702551

[91] Gaykalova DA Mambo E Choudhary A Houghton J Buddavarapu K Sanford T Novel insight into mutational landscape of head and neck squamous cell carcinoma. PLoS One. (2014) 9(3):e93102. 10.1371/journal.pone.0093102 24667986 PMC3965530

[92] India Project Team of the International Cancer Genome Consortium. Mutational landscape of gingivo-buccal oral squamous cell carcinoma reveals new recurrently-mutated genes and molecular subgroups. Nat Commun. (2013) 4:2873. 10.1038/ncomms3873 24292195 PMC3863896

[93] Rotimi SO Rotimi OA Salhia B. A review of cancer genetics and genomics studies in Africa. Front Oncol. (2021) [cited 2022 Aug 4] 10. 10.3389/fonc.2020.606400 33659210 PMC7917259

[94] Mossey PA Modell B. Epidemiology of oral clefts 2012: an international perspective. Cleft Lip Palate. (2012) 16:1–18. 10.1159/000337464 22759666

[95] Conradie EH Malherbe H Hendriksz CJ Dercksen M Vorster BC. An Overview of Benefits and Challenges of Rare Disease Biobanking in Africa, Focusing on South Africa. Biopreservation Biobanking. (2021 Apr 19) [cited 2022 Jul 23]. Available from: https://www.liebertpub.com/doi/10.1089/bio.2020.0108 10.1089/bio.2020.0108 33567219

[96] Lumaka A Carstens N Devriendt K Krause A Kulohoma B Kumuthini J Increasing African genomic data generation and sharing to resolve rare and undiagnosed diseases in Africa: a call-to-action by the H3Africa rare diseases working group. Orphanet J Rare Dis. (2022) 17(1):230. 10.1186/s13023-022-02391-w 35710439 PMC9201791

[97] Nguengang Wakap S Lambert DM Olry A Rodwell C Gueydan C Lanneau V Estimating cumulative point prevalence of rare diseases: analysis of the Orphanet database. Eur J Hum Genet. (2020) 28(2):165–73. 10.1038/s41431-019-0508-0 31527858 PMC6974615

[98] Yang G Cintina I Pariser A Oehrlein E Sullivan J Kennedy A. The national economic burden of rare disease in the United States in 2019. Orphanet J Rare Dis. (2022) 17(1):163. 10.1186/s13023-022-02299-5 35414039 PMC9004040

[99] Angelis A Tordrup D Kanavos P. Socio-economic burden of rare diseases: a systematic review of cost of illness evidence. Health Policy Amst Neth. (2015) 119(7):964–79. 10.1016/j.healthpol.2014.12.016 25661982

[100] Butali A Adeyemo WL Mossey PA Olasoji HO Onah II Adebola A Prevalence of orofacial clefts in Nigeria. Cleft Palate Craniofac J. (2014) 51(3):320–5. 10.1597/12-135 23557093 PMC3706513

[101] El-Shazly M Helmy Y Abdelsalam L Ali T. Global incidence of cleft palate. In: Fayyaz GQ, editors. Surgical atlas of cleft palate and palatal fistulae. Singapore: Springer (2020) [cited 2022 Jul 22]. p. 1–6. Available from: 10.1007/978-981-15-3889-6_129-1

[102] Muenke M Adeyemo A Kruszka P. An electronic atlas of human malformation syndromes in diverse populations. Genet Med. (2016) 18(11):1085–7. 10.1038/gim.2016.3 26938780

[103] Tekendo-Ngongang C Dahoun S Nguefack S Gimelli S Sloan-Béna F Wonkam A. Challenges in clinical diagnosis of williams-beuren syndrome in sub-Saharan Africans: case reports from Cameroon. Mol Syndromol. (2014) 5(6):287–92. 10.1159/000369421 25565928 PMC4281575

[104] Lumaka A Cosemans N Lulebo Mampasi A Mubungu G Mvuama N Lubala T Facial dysmorphism is influenced by ethnic background of the patient and of the evaluator. Clin Genet. (2017) 92(2):166–71. Available from: http://onlinelibrary.wiley.com/doi/abs/10.1111/cge.12948 27925162 10.1111/cge.12948

[105] Vorster A Beighton P Chetty M Ganie Y Henderson B Honey E Osteogenesis imperfecta type 3 in South Africa: causative mutations in FKBP10. S Afr Med J. (2017) 107(5):457–62. 10.7196/SAMJ.2017.v107i5.9461 28492130

[106] Chetty M Roberts T Shaik S Beighton P. Dentinogenesis imperfecta in Osteogenesis imperfecta type XI in South Africa: a genotype–phenotype correlation. BDJ Open. (2019) 5(1):1–5. 10.1038/s41405-019-0014-z 30993005 PMC6459848

[107] Tekendo-Ngongang C Agenbag G Bope CD Esterhuizen AI Wonkam A. Noonan syndrome in South Africa: clinical and molecular profiles. Front Genet. (2019) [cited 2022 Jul 27] 10. 10.3389/fgene.2019.00333 31057598 PMC6477999

[108] Quach H Quintana-Murci L. Living in an adaptive world: genomic dissection of the genus Homo and its immune response. J Exp Med. (2017) 214(4):877–94. 10.1084/jem.20161942 28351985 PMC5379985

[109] Schlebusch CM Malmström H Günther T Sjödin P Coutinho A Edlund H Southern African ancient genomes estimate modern human divergence to 350,000 to 260,000 years ago. Science. (2017) 358(6363):652–5. 10.1126/science.aao6266 28971970

[110] Adeola HA Adefuye A Soyele O Butali A. The dentist-scientist career pathway in Africa: opportunities and obstacles. Korean J Med Educ. (2018) 30(3):189–98. 10.3946/kjme.2018.93 30180506 PMC6127611

[111] Roberts T Yip WK Mofokeng LN Hijarunguru N Ngaxa K Mathura T Genetics in oral health: the need for human genetics in the dentistry curriculum. South Afr Dent J. (2018) 73(4):271–2. Available from: http://www.scielo.org.za/scielo.php?script=sci_abstract/pid=S0011-85162018000400024/lng=en/nrm=iso/tlng=es

[112] Pennisi E. Upstart DNA sequencers could be a “game changer”. Science. (2022) 376(6599):1257–8. 10.1126/science.add4867 35709273

[113] World Health Organization. Accelerating access to genomics for global health: Promotion, implementation, collaboration, and ethical, legal, and social issues: A report of the WHO science council. Geneva: World Health Organization (2022) [cited 2022 Sep 12]. Available from: https://apps.who.int/iris/handle/10665/359560

